# Comparative Evaluation and Performance of Large Language Models in Clinical Infection Control Scenarios: A Benchmark Study

**DOI:** 10.3390/healthcare13202652

**Published:** 2025-10-21

**Authors:** Shuk-Ching Wong, Edwin Kwan-Yeung Chiu, Kelvin Hei-Yeung Chiu, Anthony Raymond Tam, Pui-Hing Chau, Ming-Hong Choi, Wing-Yan Ng, Monica Oi-Tung Kwok, Benny Yu Chau, Michael Yuey-Zhun Ng, Germaine Kit-Ming Lam, Peter Wai-Ching Wong, Tom Wai-Hin Chung, Siddharth Sridhar, Edmond Siu-Keung Ma, Kwok-Yung Yuen, Vincent Chi-Chung Cheng

**Affiliations:** 1 Infection Control Team, Queen Mary Hospital, Hong Kong West Cluster, Hong Kong SAR, China; shchwong@hku.hk (S.-C.W.); nwy895@ha.org.hk (W.-Y.N.);; 2 School of Nursing, Li Ka Shing Faculty of Medicine, The University of Hong Kong, Pokfulam, Hong Kong SAR, China; 3 Department of Microbiology, School of Clinical Medicine, Li Ka Shing Faculty of Medicine, The University of Hong Kong, Pokfulam, Hong Kong SAR, Chinachy731@ha.org.hk (K.H.-Y.C.);; 4 Department of Microbiology, Queen Mary Hospital, Hong Kong West Cluster, Hong Kong SAR, China; 5 Department of Medicine, Queen Mary Hospital, Hong Kong West Cluster, Hong Kong SAR, China; 6 Infection Control Branch, Centre for Health Protection, Department of Health, Hong Kong SAR, China; edmond_sk_ma@dh.gov.hk

**Keywords:** infection control, large language models, evaluation

## Abstract

**Background**: Infection prevention and control (IPC) in hospitals relies heavily on infection control nurses (ICNs) who manage complex consultations to prevent and control infections. This study evaluated large language models (LLMs) as artificial intelligence (AI) tools to support ICNs in IPC decision-making processes. Our goal is to enhance the efficiency of IPC practices while maintaining the highest standards of safety and accuracy. **Methods**: A cross-sectional benchmarking study at Queen Mary Hospital, Hong Kong assessed three LLMs—GPT-4.1, DeepSeek V3, and Gemini 2.5 Pro Exp—using 30 clinical infection control scenarios. Each model generated clarifying questions to understand the scenarios before providing IPC recommendations through two prompting methods: an open-ended inquiry and a structured template. Sixteen experts, including senior and junior ICNs and physicians, rated these responses on coherence, conciseness, usefulness and relevance, evidence quality, and actionability (1–10 scale). Quantitative and qualitative analyses assessed AI performance, reliability, and clinical applicability. **Results**: GPT-4.1 and DeepSeek V3 scored significantly higher on the composite quality scale, with adjusted means (95% CI) of 36.77 (33.98–39.57) and 36.25 (33.45–39.04), respectively, compared with Gemini 2.5 Pro Exp at 33.19 (30.39–35.99) (*p* < 0.001). GPT-4.1 led in evidence quality, usefulness, and relevance. Gemini 2.5 Pro Exp failed to generate responses in 50% of scenarios under structured prompt conditions. Structured prompting yielded significant improvements, primarily by enhancing evidence quality (*p* < 0.001). Evaluator background influenced scoring, with doctors rating outputs higher than nurses (38.83 vs. 32.06, *p* < 0.001). However, a qualitative review revealed critical deficiencies across all models, for example, tuberculosis treatment solely based on a positive acid-fast bacilli (AFB) smear without considering nontuberculous mycobacteria in DeepSeek V3 and providing an impractical and noncommittal response regarding the de-escalation of precautions for *Candida auris* in Gemini 2.5 Pro Exp. These errors highlight potential safety risks and limited real-world applicability, despite generally positive scores. **Conclusions**: While GPT-4.1 and DeepSeek V3 deliver useful IPC advice, they are not yet reliable for autonomous use. Critical errors in clinical judgment and practical applicability highlight that LLMs cannot replace the expertise of ICNs. These technologies should serve as adjunct tools to support, rather than automate, clinical decision-making.

## 1. Introduction

In Hong Kong, infection prevention and control (IPC) is a critical clinical service in hospitals, predominantly supported by a limited number of ICNs who work to prevent healthcare-associated infections and outbreaks [1,2,3,4]. ICNs carry out multiple responsibilities including clinical audits, surveillance, outbreak control, and managing a high volume of consultations, ranging from bedside clinical inquiries to phone and email consultations. These consultations, often complex, require in-depth clinical analysis and decision making. Junior or less experienced ICNs, in particular, may face challenges in making timely and accurate infection control decisions. Therefore, innovative measures that can reduce workload and improve clinical efficiency are critically needed.

Prior approaches to IPC have utilized various strategies such as computerized surveillance systems [5,6], predictive analytics [7,8], antimicrobial stewardship programs [9], and interventions to improve compliance with infection control practices like hand hygiene [10,11]. Recently, artificial intelligence (AI) technologies have shown transformative potential in enhancing IPC [12,13], especially evidenced by their impact on preparedness and response during the COVID-19 pandemic [14,15,16].

Large language models (LLMs), a subset of AI, have recently been evaluated for their potential roles in clinical decision support including triage, referral, and diagnosis workflows [17]. Despite their promise, current AI models, including LLMs, require human oversight to ensure safety and accuracy in clinical infectious disease consultations [18]. In this study, we evaluated three leading LLMs for their capacity to support ICNs during complex IPC consultations. GPT-4.1 (OpenAI) is known for its state-of-the-art performance across a range of professional and academic examinations [19]. DeepSeek V3 (DeepSeek AI) uses a mixture-of-experts architecture optimized for domain-specific queries and is designed with the Asian context in mind, enhancing regional applicability [20]. Gemini 2.5 Pro Exp (Google DeepMind), an experimental multimodal transformer, integrates text, code, and image inputs, offering potential for complex content analysis [21]. Together, these models represent a broad range of technological approaches, enabling a comparison of their effectiveness in the specialized, high-stakes context of IPC, where both clinical accuracy and contextual understanding are critical.

Our study aims to assess whether LLMs can serve as an effective decision support tool for ICNs by addressing existing gaps in consultation support and workload management. Through this evaluation, we seek to determine whether these models can enhance the efficiency and quality of IPC practices without compromising patient safety.

## 2. Literature Review

The integration of AI into healthcare has promised to revolutionize clinical practice. Within this landscape, LLMs have emerged as a particularly powerful class of AI, capable of understanding, generating, and reasoning with complex human language. This has led to their evaluation in a variety of clinical decision support roles including patient triage, diagnostic assistance, and the summarization of electronic health records [17].

Historically, the application of AI in IPC has focused on structured data analysis. For instance, computerized surveillance systems and machine learning algorithms have been successfully employed to automate the detection of healthcare-associated infections and optimize antimicrobial stewardship programs [22,23,24,25]. The COVID-19 pandemic further accelerated the adoption of AI, where it played a crucial role in epidemiological modeling, resource allocation, and public health surveillance, highlighting its potential to enhance pandemic preparedness and response [26]. These applications, however, primarily relied on analyzing quantitative data and did not typically engage with the unstructured, dialogue-based nature of clinical consultations.

The advent of sophisticated LLMs presents a new frontier for IPC support. Unlike traditional AI, modern LLMs can process and respond to complex, open-ended clinical scenarios, mirroring the consultative work of ICNs. Preliminary evaluations of general-purpose LLMs in clinical medicine have yielded mixed results. While these models demonstrate impressive fluency and a broad knowledge base, studies have consistently raised concerns regarding factual accuracy, the risk of “hallucinations” (generating plausible but incorrect information), and a failure to grasp subtle clinical context, which can lead to potentially harmful recommendations [18,27] (Table 1).

This body of work reveals several critical gaps that our study aims to address. First, there is a scarcity of direct, head-to-head benchmark comparisons of the latest generation of LLMs, such as GPT-4.1, DeepSeek V3, and Gemini 2.5 Pro Exp, within a specialized clinical domain. Second, few studies have focused specifically on the complex, high-stakes nature of IPC consultations, which demand not only evidence-based knowledge, but also practical, actionable guidance tailored to specific institutional contexts. Third, the impact of prompt engineering, specifically the use of structured versus open-ended prompts, on the quality and safety of LLM outputs in IPC has not been rigorously evaluated. Finally, the influence of the evaluator’s professional background (e.g., physician vs. nurse) on the assessment of AI-generated advice remains an underexplored but critical area. Our study is designed to fill these gaps by providing a robust, multidisciplinary evaluation of leading LLMs in realistic IPC scenarios.

## 3. Materials and Methods

### 3.1. Setting

This study was conducted by the Infection Control Team (ICT) in collaboration with the Department of Microbiology and the Department of Medicine at Queen Mary Hospital, a tertiary referral center with a capacity of approximately 1700 beds within the Hong Kong West Cluster, under the governance of the Hospital Authority [28]. The ICT comprises a team of frontline professionals, co-supervised by an Infection Control Officer (ICO) and a Senior Nursing Officer (SNO), along with seven ICNs with varying levels of experience. The ICT is responsible for implementing hospital-wide IPC policies, conducting staff education programs, performing active surveillance of healthcare-associated infections and monitoring compliance with infection prevention protocols. Daily activities include outbreak investigations, risk assessments, review of IPC practices, and acting as a point of contact for clinical staff on issues such as hand hygiene, vaccination campaigns, and needlestick injuries. The ICT also participates in multidisciplinary meetings to discuss complex cases and develop actionable IPC strategies.

Clinical staff can consult the ICT through multiple channels: face-to-face curbside consultations in clinical areas, telephone calls, and email communications. All consultations, which vary in complexity, are thoroughly documented for retrospective review and incorporated into internal training sessions conducted twice weekly. This structured workflow ensures timely and evidence-based responses to IPC concerns and supports continuous quality improvement within the hospital setting.

### 3.2. Study Design

This is a cross-sectional benchmarking study involving three LLMs: GPT-4.1 (version gpt-4.1-2025-04-14; OpenAI, San Francisco, CA, USA), DeepSeek V3 (version DeepSeek-V3-0324; Hangzhou DeepSeek Artificial Intelligence Basic Technology Research Co. Ltd., Hangzhou, China), and Gemini 2.5 Pro Experimental (Gemini 2.5 Pro Exp; version gemini-2.5-pro-exp-03-25, Google DeepMind, London, UK). All interactions with the LLMs were conducted via the Poe platform (version 1.1.32; Quora, Inc., Mountain View, CA, USA). All models were run using the platform’s default settings for temperature and maximum tokens to evaluate out-of-the-box performance.

The study utilized 30 clinical infection control scenarios derived from the real-world consultation records of experienced ICNs (Table S1). The scenarios were selected to reflect a broad range of IPC concerns, encompassing various pathogens, clinical presentations, and management challenges. Inclusion criteria required scenarios to be representative of typical clinical queries encountered by ICNs, while duplicate or highly similar cases were excluded to maintain diversity. We balanced the selection across topics such as outbreak management, exposure risk management, multidrug-resistant organisms (MDROs), and disinfection protocols. Each scenario’s complexity and urgency were reviewed qualitatively by infection control experts to ensure relevance and real-world applicability.

To protect patient confidentiality, all scenarios were thoroughly de-identified according to institutional protocols before use. While a unique “reference answer” for each scenario was not provided due to variability in clinical practice shaped by local epidemiology and institutional policies, all scenarios underwent expert review to confirm clinical appropriateness. This approach acknowledges the inherent variability in IPC decision making while enabling a robust, realistic evaluation of LLM capabilities in supporting ICNs.

Each of the 30 scenarios was independently input into each of the three LLMs. Each LLM was prompted to generate clarifying questions it would ask to better understand the scenario before providing recommendations. This resulted in one set of questions from each LLM for each scenario.

The key panel consisting of an SNO (author: S.-C.W.) and two clinical microbiologists (authors: E.K.-Y.C. and K.H.-Y.C.) reviewed the LLM-generated questions and provided standardized answers for each set of questions corresponding to each LLM and scenario. For each LLM and scenario, the original scenario text, the specific questions generated by that LLM, and the expert-provided answers were compiled. This compiled information was then re-input into the respective LLM to generate IPC recommendations using two distinct methods: an open-ended question with direct prompt of ‘Based on the provided scenario and the answers to your questions, what are your infection control recommendations?’ and a structured prompt template, as illustrated in Table 2. The structured template employed a multistep, two-phase prompting strategy. This approach first required the LLM to generate clarifying questions (Phase 1), which were then answered by an expert panel. Subsequently, the LLM was prompted again with the complete information to formulate its final recommendations (Phase 2), thereby guiding it through a more deliberative reasoning process. If an LLM failed to produce a response for a given scenario, the scenario was resubmitted. The precise retry logic was to attempt generation up to three times. If the LLM failed to generate an output after three attempts, the response for that data point was recorded as a failure and replaced with the text, “Failed to run despite multiple attempts, please skip this section” for the subsequent evaluation phase. The time taken by the key panel to process the clinical infection control scenarios, from reviewing the LLM-generated questions to completing the infection control recommendations using both methods described above for each LLM, was recorded, resulting in 180 unique outputs (each comprising questions and recommendations). However, as detailed in Section 4, 15 generation failures were recorded for the Gemini 2.5 Pro Exp, reducing the total number of successful outputs to 165.

To ensure objective assessments, all evaluators (except E.K.-Y.C. who managed the data compilation) were blinded to the identity of the LLMs that generated each output. The evaluation was conducted using the Qualtrics survey platform (Qualtrics, Provo, UT, USA), a web-based tool for creating, distributing, and analyzing surveys. All identifiers related to the specific LLM were removed from the outputs. To mitigate potential order bias during the evaluation, the sequence of the LLM-generated responses was randomized for each clinical scenario [29]. To promote consistent application of the rating scale, all evaluators were provided with the detailed rubric in Table 3, which defines the characteristics of low (1) and high (10) scores for each criterion.

**Figure 1:**
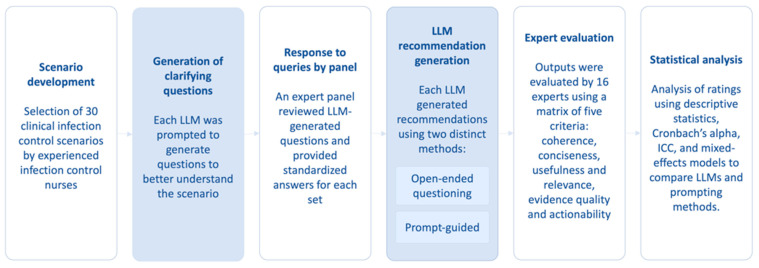
Workflow of a cross-sectional benchmarking study involving LLMs for clinical infection control scenarios.

Each of these 165 output sets was evaluated by a group of sixteen human evaluators, consisting of eight ICNs, with four who classified as senior nurses (advanced practice nurses or SNO) and four as junior nurses (registered nurses) as well as eight medical doctors including clinical microbiologists, infectious disease physicians, or public health experts. Among the doctors, four were classified as senior doctors (associate consultants, associate professor or above), while four were classified as junior doctors (residents). Each evaluator independently rated each output (Figure 1). Each output (both questions and recommendations) was evaluated using a standardized matrix of five criteria: coherence, conciseness, usefulness and relevance, evidence quality, and actionability, as previously described with modifications [18]. Each criterion was rated on a scale from 1 to 10. Therefore, a total of 2640 individual rating instances were collected for the primary outcome measures, which represent the mean expert rating for each LLM output (questions and recommendations) across the five criteria. Secondary outcome measurements included the internal consistency of the rating scale, inter-rater reliability for each criterion and overall, and a composite overall quality score for each output.

Following the quantitative analysis, a qualitative review of the LLM-generated recommendations was conducted using a thematic analysis approach to identify deficiencies. This analysis focused on identifying common themes related to errors in clinical judgment, factual accuracy, and the practical applicability of the advice in a real-world hospital setting. Representative examples were compiled to illustrate their shortcomings.

### 3.3. Study Hypothesis and Objectives

We hypothesized that the quality of questions and recommendations generated by different LLMs may vary significantly in response to clinical infection control scenarios. Furthermore, we expected that using a structured prompt template for generating recommendations would result in higher-quality outputs compared with an open-ended question approach across all evaluated LLMs. The primary objective of this study was to compare the quality of questions and recommendations generated by three leading LLMs in response to infection control scenarios, assessed across multiple dimensions including coherence, conciseness, usefulness and relevance, evidence quality, and actionability. While it is recognized that effective prompt structuring can enhance LLM responses in general contexts, the secondary objective was to evaluate whether a structured prompt template improved the quality of the LLM outputs, specifically within the complex and high-stakes domain of IPC. This study fills a critical gap by providing domain-specific validation of prompt design, benchmarking multiple state-of-the-art models with clinically relevant metrics and expert evaluation, thereby offering important insights into the practical application of LLMs as decision support tools in IPC practice.

### 3.4. Statistical Analysis

Descriptive statistics were presented as the mean ± standard deviation (SD). The internal consistency of the five-item rating scale was assessed using Cronbach’s alpha and corrected item-total correlations. An exploratory factor analysis (EFA) using principal axis factoring was conducted to assess the underlying structure of the five criteria. Inter-rater reliability for each criterion was assessed using the intraclass correlation coefficient (ICC).

The composite score for each LLM-generated output was calculated as the sum of the five individual criterion scores. The primary analysis for the baseline comparison (open-ended question approach) utilized linear mixed-effects models to compare the mean scores for each criterion. In these models, the LLM was specified as a fixed effect, while both evaluator and scenario were included as crossed random intercepts. This model structure allowed us to simultaneously account for the variability introduced by different evaluators’ rating styles and by the inherent differences in complexity or focus across the 30 clinical scenarios. Variance components were calculated from each model to quantify the proportion of total variance attributable to the fixed and random effects. Post hoc pairwise comparisons of the estimated marginal means between LLMs were conducted with a Bonferroni adjustment for multiple comparisons. Finally, Kruskal–Wallis H tests with Bonferroni-corrected pairwise comparisons were performed as a sensitivity analysis.

Subsequently, to assess the effect of the structured prompt template, a separate set of mixed-effects models was constructed using the data from both prompting conditions. These models included the prompting method (open-ended question vs. structured prompt template), the LLM, and the interaction between these two factors as fixed effects. The evaluator was included as a random intercept. Sensitivity analysis was conducted on the subset of data excluding the 15 scenarios where generation failures occurred. Where significant main or interaction effects were observed, post hoc pairwise analyses were conducted to further explore group differences.

The influence of evaluator characteristics (profession and seniority) on scoring was also examined using mixed-effects models, with appropriate fixed and random effects. Statistical significance was set at *p* < 0.05 for all analyses. All statistical analyses were performed using IBM SPSS Statistics version 31.0.0.0 (117).

## 4. Results

### 4.1. Pre-Evaluation of LLMs to Clinical Infection Control Scenarios

Thirty representative and nonduplicate scenarios were selected by the co-supervisor of the ICT. The three key panel members collaborated closely to engage with the three LLMs, ensuring a thorough interaction regarding the 30 selected scenarios. Each scenario was processed in sequence by GPT-4.1, DeepSeek V3, and Gemini 2.5 Pro Exp. There was no statistical difference in the time required to process each clinical infection control scenario by GPT-4.1, DeepSeek V3, and Gemini 2.5 Pro Exp, which were 3 ± 1 min, 2 ± 1 min, and 2.5 ± 2 min, respectively (*p* = 0.102, Kruskal–Wallis test). All three LLMs provided responses to the open-ended question and the structured prompt template, except for Gemini 2.5 Pro Exp, which failed to execute the structured prompt in 15 (50%) out of 30 scenarios.

### 4.2. Internal Consistency and Reliability Analysis

The five-item rating scale demonstrated excellent internal consistency across all evaluations (*N* = 2640) with a Cronbach’s alpha of 0.932, as detailed in Table S2. The corrected item-total correlations for each criterion were high, ranging from 0.742 to 0.884, indicating that all items contributed effectively to the composite score.

In addition to internal consistency, we examined dimensionality using EFA. An EFA confirmed the suitability of the data for analysis (Kaiser–Meyer–Olkin = 0.896; Bartlett’s test of sphericity, *p* < 0.001). The EFA extracted a single factor that accounted for 79.6% of the total variance, with all five criteria exhibiting strong factor loadings (factor loadings > 0.76). These results support treating the five items as indicators of a single latent construct and justify the use of the composite score.

Inter-rater reliability was quantified using a two-way random-effects, consistency, average-measures ICC, ICC (2,16), with 95% CIs derived from the F distribution. Using Koo and Li’s interpretive thresholds [30], agreement was good for conciseness (ICC = 0.815; 95% CI: 0.781–0.846) but moderate for evidence quality (0.735; 0.692–0.774) and actionability (0.533; 0.474–0.588). Agreement was poor for usefulness and relevance (0.478; 0.416–0.536) and coherence (0.411; 0.346–0.472) (Table S3).

### 4.3. Descriptive Statistics

Descriptive statistics for each LLM are summarized in Table S4. Overall, GPT-4.1 and DeepSeek V3 achieved higher unadjusted mean scores across most criteria compared with Gemini 2.5 Pro Exp including composite score (GPT-4.1: mean ± SD, 36.77 ± 7.53; DeepSeek V3: 36.25 ± 8.02; Gemini 2.5 Pro Exp: 33.22 ± 7.92).

### 4.4. Analysis of Variance Components

Variance components were calculated from the linear mixed-effects models (Table S5, which revealed that a substantial portion of the total variance across all criteria was attributable to the evaluators, ranging from 29.9% for conciseness to 47.8% for coherence. The residual (within-group) variance accounted for between 50.7% and 68.9%. The specific clinical scenario contributed minimally to the overall variance, accounting for less than 3% across all dependent variables. This indicates that the individual rating styles and interpretations of the evaluators were a much more significant driver of score variation than the differences between the 30 clinical scenarios, reinforcing the necessity of including evaluator as a random effect in the model.

### 4.5. Pairwise Comparison of LLMs Under Open-Ended Question

Pairwise comparisons using mixed-effects models revealed consistent and statistically significant differences among the models. For the composite quality score, both GPT-4.1 (mean difference [MD] = 3.58, 95% CI 2.88–4.28, *p* < 0.001) and DeepSeek V3 (MD = 3.06, 95% CI 2.36–3.75, *p* < 0.001) significantly outperformed Gemini 2.5 Pro Exp. In contrast, there was no statistically significant difference between GPT-4.1 and DeepSeek V3 on the composite score (MD = 0.52, 95% CI −0.12–1.17, *p* = 0.153).

**Figure 2:**
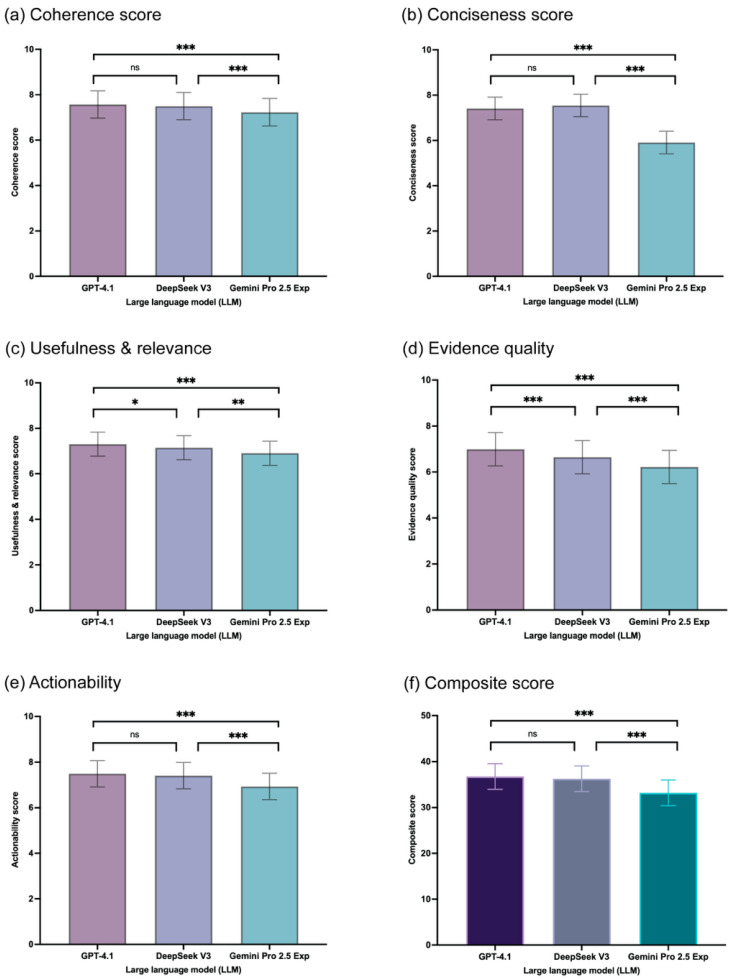
Comparison of estimated marginal mean composite scores among the LLMs. Bars represent the estimated marginal means derived from the mixed-effects model. Significance levels: * *p* < 0.05; ** *p* < 0.01; *** *p* < 0.001; ns = not significant.

This pattern of GPT-4.1 and DeepSeek V3 outperforming Gemini 2.5 Pro Exp was consistent across all individual criteria (*p* ≤ 0.002 for all comparisons). When comparing GPT-4.1 and DeepSeek V3 directly, GPT-4.1 scored significantly higher on usefulness and relevance (MD = 0.16, *p* = 0.048) and evidence quality (MD = 0.35, *p* < 0.001). No significant differences were found between the two models for coherence, conciseness, or actionability (Figure 2, Table S6). A sensitivity analysis using the Kruskal–Wallis H test with Bonferroni-corrected pairwise comparisons confirmed these primary findings, revealing a similar pattern of significant differences among the models (Table S7).

### 4.6. Effect of Structured Prompting

The analysis of the structured prompt’s effect revealed that this approach led to a small but statistically significant improvement in the composite score across all LLMs. While the magnitude of this overall improvement did not differ significantly between the models, the primary benefit was a significant enhancement in “evidence quality” (*p* < 0.001). Notably, the degree of improvement for this specific criterion varied significantly among the LLMs. In contrast, structured prompting did not confer significant benefits for the other criteria (coherence, conciseness, usefulness and relevance, and actionability) (Table 4). A sensitivity analysis excluding the 15 scenarios with failed generations yielded a consistent pattern of results, confirming the robustness of these findings (Table S8).

### 4.7. Influence of Evaluator Characteristics

Analysis of the evaluator characteristics, summarized in Table 5, showed that doctors assigned significantly higher adjusted mean composite scores than the nurses (38.83 vs. 32.06, *p* < 0.001). Among the doctors, senior evaluators gave higher scores than junior colleagues (42.42 vs. 35.23, *p* < 0.001), whereas there was no significant difference between senior and junior nurses (32.08 vs. 32.03, *p* = 0.915).

### 4.8. Qualitative Analysis of Deficiencies

Beyond the quantitative scores, our qualitative review identified critical deficiencies across all three LLMs. Common themes included poor clinical judgment such as recommending aggressive measures for a low-suspicion case and providing factually incorrect advice that contradicted standard infection control policies, particularly regarding multidrug-resistant *Acinetobacter* species (MDRA) isolation. Other recurring issues were a lack of real-world practicality, with responses being overly academic or failing to prioritize urgent actions. These findings, summarized in Table 6, reveal that even the higher-performing models may generate clinically significant and potentially unsafe recommendations.

## 5. Discussion

In this study, we demonstrated the applicability of LLMs in clinical infection control scenarios. Our primary findings indicate that GPT-4.1 and DeepSeek V3 consistently delivered a superior and largely comparable performance across most evaluation metrics when compared with Gemini 2.5 Pro Exp, which demonstrated significantly lower performance across all domains. While GPT-4.1 and DeepSeek V3 showed similar strengths in coherence, conciseness, and actionability, GPT-4.1 exhibited a distinct advantage in evidence quality. Furthermore, our results indicate that using a structured prompt yielded a small but statistically significant improvement in the overall quality of recommendations, particularly in terms of evidence quality.

However, the reliability of these models varied notably. Gemini 2.5 Pro Exp demonstrated a 50% failure rate when executing the structured prompt, primarily due to its tendency to produce much longer outputs compared with the other models. A plausible technical explanation is that the combined length of the detailed input prompt and the model’s characteristically verbose output may have exceeded the usable context window on the platform, contributing to failures. However, we did not collect token-level diagnostics or platform error logs to verify this, so this interpretation remains speculative. The observed performance differences may be linked to the underlying architecture and training data of each model. The superior evidence quality of GPT-4.1 observed in this study, for instance, may reflect its training on extensive biomedical datasets. Similarly, DeepSeek V3’s mixture-of-experts architecture, which optimizes for specialized tasks, likely contributed to its stable and high-quality outputs [31]. Conversely, Gemini 2.5 Pro Exp’s high failure rate and lower scores could be attributed to its experimental nature and focus on multimodality (e.g., integrating text, code, and images). Such multimodal strengths offer little advantage in this study’s text-based scenarios and may have contributed to its characteristically verbose outputs, potentially exceeding the platform’s maximum context window and causing generation failures.

Our study benchmarked three leading LLMs from distinct global developers (OpenAI, DeepSeek, and Google DeepMind) to provide a robust and diverse comparison. This approach enabled an assessment across different development philosophies and potential training data biases. GPT-4.1 is widely recognized as a high-performing industry benchmark, making its inclusion essential for context. DeepSeek V3 represents an advanced LLM developed specifically for the Asian market, allowing for the assessment of its global relevance. The experimental version of the Gemini 2.5 Pro model was included to appraise the latest advancements in LLM technology, despite its increased instability and higher task failure rate. Importantly, these three readily accessible models represent the current state-of-the-art in LLM technology, making our findings relevant to contemporary clinical practice and highlighting key performance differences among leading platforms.

A key aspect of our study was the composition of our evaluation panel and the influence of their professional backgrounds on scoring outcomes. Unlike larger questionnaire-based studies involving extensive participant pools [32,33], we prioritized the quality of responses by involving a selected group of evaluators, comprising ICNs, clinical microbiologists, infectious disease physicians, and public health experts. Our approach resulted in 825 criterion-level responses per evaluator, a substantially greater workload per participant compared with typical focus group studies. For context, a review of 220 papers published across 117 journals reported a median focus group size of five participants [34]. Our balanced group, with an equal representation of ICNs and doctors and a mix of junior and senior ranks, revealed notable scoring differences. Notably, medical doctors awarded significantly higher overall scores than ICNs, potentially because doctors focused on general consistency with the clinical guidelines, while ICNs assessed the outputs more critically for practical implementation. Furthermore, senior physicians rated outputs significantly higher than their junior colleagues, perhaps reflecting a greater appreciation for the underlying clinical reasoning, whereas junior physicians may have focused more on strict protocol adherence. In contrast, scores between junior and senior nurses were closely aligned, likely reflecting standardized training. These findings align with our previous studies reporting that clinical experience and domain expertise influenced the clinicians’ interpretation of AI-generated outputs [18].

Beyond the quantitative metrics, our qualitative review uncovered important shortcomings in the outputs related to clinical judgement and real-world practicality, as detailed in Table 6. For example, Gemini 2.5 Pro Exp provided a confusing and impractical response regarding *Candida auris* de-escalation, while DeepSeek V3 suggested immediate tuberculosis treatment based solely on a positive AFB smear, overlooking alternatives such as nontuberculous mycobacteria. Even the better performing GPT-4.1 was not immune to error, incorrectly advising against single-room isolation for a patient colonized with MDRA, a recommendation that contradicts standard infection control policy. These examples highlight that while these models can effectively organize information, they may fall short in delivering clinical reasoning and the risk assessment needed for infection control practice.

The theoretical implications of our study include advancing knowledge about the variability in performance of contemporary LLMs within the specialized clinical domain of infection control, illuminating how prompt structuring can quantitatively enhance the output quality and demonstrating the critical influence of evaluator expertise on AI output assessment. These findings contribute to the broader understanding of AI model behavior in applied clinical contexts and support the need for domain-specific validation when adapting general AI technologies to healthcare.

**Figure 3:**
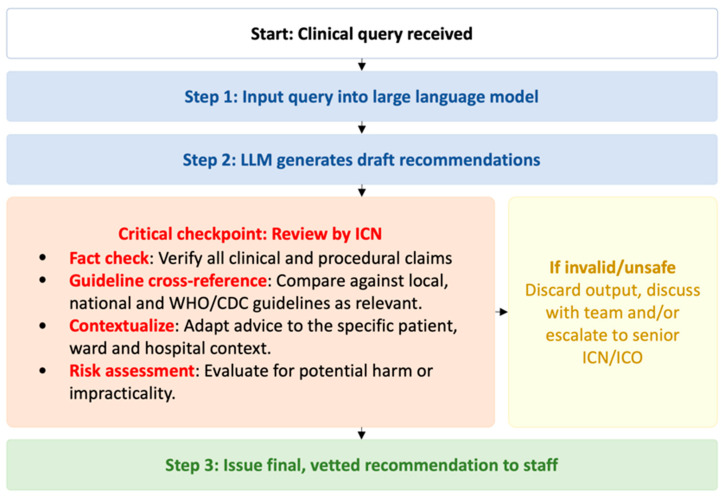
Proposed human-in-the-loop workflow for the safe integration of LLMs in clinical infection control. Abbreviations: ICN, infection control nurse; ICO, infection control officer.

The practical implications for IPC workflows are significant. Our results emphasize that while LLMs can generate actionable guidance, they currently lack the reliability and clinical judgement required to replace ICNs. Instead, these tools should be integrated as decision support adjuncts to augment expert judgement, reduce workload, and enhance efficiency. To implement this safely and effectively, we propose a structured human-in-the-loop workflow (Figure 3) where the ICN acts as the final clinical reviewer. This model positions the LLM as a tool for generating initial draft recommendations, which must then undergo a mandatory critical review. This checkpoint involves four essential actions: (1) fact-checking all clinical and procedural claims, (2) cross-referencing against relevant local and international guidelines, (3) contextualizing the advice to the specific patient and hospital setting, and (4) conducting a risk assessment for potential harm or impracticality. By adopting such a framework, which includes a clear escalation pathway for unsafe outputs, healthcare institutions can leverage the efficiency of LLMs while providing the necessary professional oversight. This approach not only ensures patient safety but also provides a transparent reference for guiding the selection and ongoing improvement of AI tools for clinical needs.

Given the modest but significant gains observed with structured prompting, particularly for evidence quality, future work should evaluate whether more advanced prompting strategies can yield further improvements. Building on the structured zero-shot baseline established in this study, future research should explore advanced prompting techniques such as one-shot, few-shot, and chain-of-thought prompting. Such investigations would be valuable for determining whether these methods can further enhance the reliability and accuracy of LLM recommendations in complex clinical scenarios, ultimately moving these tools closer to safe and effective implementation.

Ultimately, the collaboration of AI systems and specialized clinical staff like ICNs promise to improve infection control outcomes and foster a more efficient, informed healthcare environment, provided that AI is applied thoughtfully and with a full awareness of its current limitations.

This study had several limitations. First, it relied on 30 clinical infection control scenarios deemed representative by the ICNs. While these scenarios encompassed a variety of topics, including epidemiologically important viruses, MDROs, contact tracing, outbreak management, disinfection, exposure risk, post-exposure management, and risk assessment across diverse clinical presentations, they may not have captured the full complexity and variability of real-world clinical situations. Second, the evaluation criteria were inherently subjective. To mitigate this, we provided clear definitions of these criteria to our invited evaluators, who were practicing ICNs and medical specialists, and we believe these participants performed the assessments conscientiously. Third, the findings may not be generalizable to other healthcare settings or regions with different infection control practices and challenges. Fourth, our findings are specific to the performance of the three LLMs evaluated and may not extend to all current or future models. Due to constraints in accessibility and resources, we were unable to include specialized medical LLMs such as Med-Gemini or SM70, which may offer improved reliability and accuracy for IPC. Future studies may include such specialized models for a more comprehensive evaluation. 

## 6. Conclusions

This cross-sectional benchmarking study revealed that while leading LLMs such as GPT-4.1 and DeepSeek V3 can generate actionable infection control advice, they are not yet reliable enough in autonomous use. Our qualitative analysis identified critical errors in clinical judgment and practical application across all models, suggesting that in their current state, they cannot replace the sophisticated expertise of ICNs. The optimal role for these technologies is as a powerful adjunct to augment, not automate, clinical decision-making. This study’s major contributions include providing a comprehensive, head-to-head comparison of state-of-the-art LLMs in infection control, demonstrating the benefits of structured prompting to enhance recommendation quality, and revealing how evaluator background and experience influence AI output assessments. By identifying current limitations in clinical reasoning with LLM outputs, we emphasize the indispensable role of ICNs and the potential of AI to support, rather than replace, expert decision-making. These findings offer valuable insights to inform the future development, evaluation, and integration of AI tools in IPC practice.

## Figures and Tables

**Table 1:** Summary of the key literature on AI and LLMs in clinical decision support for infection disease management and prevention.

Study (Author, Year)	Clinical Task	Key Contribution	Gap Addressed by Our Study
Wang et al. (2024) [23]; Goodman et al. (2022) [24]; Barchitta et al. (2021) [25]	HAI prediction and antimicrobial, stewardship	Demonstrated that traditional ML can automate risk stratification and optimize stewardship using structured clinical data (e.g., lab values, patient history).	Relies on structured data; not designed to handle the unstructured, dialogue-based nature of real-time clinical consultations.
Heidari et al. (2022) [26]	COVID-19 outbreak management	Provided a broad overview of AI’s utility in epidemiological modeling, diagnosis, and resource allocation during a pandemic.	High-level review; does not benchmark the performance of specific models for granular, case-based clinical decision-making in IPC.
Gaber et al. (2025) [17]	Triage, referral, and diagnosis	Benchmarked LLMs for general clinical decision support, showing they can assist in triage and diagnosis but performance varies.	Focuses on general emergency medicine; not specific to field of IPC.
Chiu et al. (2025) [18]; Schwartz et al. (2024) [27]	Infectious disease consultations	Raised critical safety alarms. Highlighted the risk of factual inaccuracies (“confabulations”) and the need for expert oversight to prevent patient harm.	Identified the problem of safety and reliability but did not: (1) perform a head-to-head benchmark of the latest models, or (2) systematically test mitigation strategies like structured prompting.

Abbreviations: AI, artificial intelligence; HAI, hospital-acquired infection; IPC, infection prevention and control; LLMs, large language models; ML, machine learning.

**Table 2:** Prompt template for inputting infection control scenarios input to the LLMs.

Introduction
You are an infection control officer in Hong Kong responsible for handling clinical queries regarding infection control. Your task is to carefully review the provided clinical scenario and obtain any missing details before formulating recommendations.
Phase 1: Clarification and information gathering
## Do not formulate recommendations in phase 1 of your response. 1. [The specific clinical scenario text (1 of 30) is inserted here.] 2. After you have provided the clarification questions, you will then receive answers to proceed to phase 2.
Phase 2: Formulating recommendations
## This phase should only begin after you have received responses to your clarification questions. 1. Begin by summarizing the enquiry in two concise sentences that capture the key context and needs of the scenario. 2. Create a table that categorizes the necessary actions by each stakeholder. The table should include these columns: Stakeholder: Define roles such as Clinical Staff, Infection Control Team, Laboratory, Hospital Management, etc. Action/Intervention Required: Clearly outline what needs to be done. Responsible Party: Specify who will take the action, if different from the stakeholder. Timeline/Deadline: Indicate when the action should be completed. Notes/Comments: Provide additional details or reference guidelines (e.g., CDC, WHO). 3. Additional guidelines: Include a brief explanation for important recommendations and reference any relevant guidelines (e.g., CDC, WHO) or established protocols that support the recommendations Ensure your recommendations are clear, actionable, and prioritized based on the risk assessment. Clearly state any assumptions made or highlight any areas where further data may be required to finalize recommendations.

Abbreviations: CDC, Centers for Disease Control and Prevention; LLMs, large language models; WHO, World Health Organization.

**Table 3:** Evaluation criteria rated on a scale from 1 to 10 including coherence, conciseness, usefulness and relevance, evidence quality, and actionability.

Criterion	Definition	Low Score (1) Represents	High Score (10) Represents
Coherence	The quality of the writing being logical, consistent, and easy to understand. The text should be well-structured and free from contradictions.	The output is confusing, illogical, internally inconsistent, or very difficult to read and understand.	The output is exceptionally clear, logical, and well-structured. The reasoning flows naturally and is easy to follow.
Conciseness	The output is concise and to the point, avoiding irrelevant information, redundancy, or excessive verbosity.	The output is overly long, repetitive, and contains significant irrelevant information that detracts from the main points.	The output is perfectly concise. The core message is delivered efficiently and without unnecessary filler.
Usefulness and relevance	The output directly addresses the core clinical problem presented in the scenario and provides information that is pertinent and helpful to an infection control professional.	The output is irrelevant to the scenario, misses the key infection control issues, or provides generic information of no practical use.	The output is highly relevant, directly addresses the critical aspects of the scenario, and provides genuinely useful insights for an infection control nurse.
Evidence quality	The degree to which the recommendations or the reasoning behind the questions align with established infection control principles, clinical guidelines (e.g., WHO, CDC), and sound scientific reasoning.	The output contains factually incorrect information, contradicts established guidelines, or provides advice that is not based on sound clinical evidence (“hallucination”).	The recommendations are fully aligned with best practices and established evidence-based guidelines. The reasoning is clinically and scientifically sound.
Actionability	The recommendations are clear, specific, practical, and can be realistically implemented by an infection control nurse in a hospital setting. The steps are well-defined.	The recommendations are vague, abstract, impractical, or lack the specific details needed for implementation.	The recommendations are concrete, specific, and provide clear, step-by-step instructions that an infection control nurse could immediately act upon.

**Table 4:** Effect of structured prompting on large language model scoring.

			Large Language Model		*p*-Value	
		GPT-4.1	DeepSeek V3	Gemini 2.5 Pro Exp	Effect of Prompt	Interaction Effect
Mean change in scoring effect of prompt	Coherence	−0.013 (−0.161, 0.136)	+0.010 (−0.138, 0.159)	+0.004 (−0.178, 0.187)	0.991	0.987
	Conciseness	−0.206 (−0.384, −0.028)	−0.123 (−0.301, 0.055)	−0.040 (−0.257, 0.178)	0.066	0.613
	Usefulness and relevance	+0.060 (−0.123, 0.244)	+0.119 (−0.064, 0.302)	+0.092 (−0.133, 0.316)	0.193	0.933
	Evidence quality	+0.765 (0.575–0.954)	+1.106 (0.916, 1.296)	+0.425 (0.193, 0.657)	<0.001	0.003
	Actionability	−0.046 (−0.221, 0.129)	+0.017 (−0.158, 0.192)	+0.052 (−0.162, 0.266)	0.913	0.846
	Composite score	+0.560 (−0.194, 1.315)	+1.129 (0.375, 1.884)	+0.533 (−0.391, 1.457)	0.019	0.661

Values represent the mean change with 95% CI in score when using a structured prompt compared with an open-ended question. *p*-values show the significance of the main effect of prompting and the interaction effect between prompt type and LLM, as derived from the mixed-effects models.

**Table 5:** Influence of evaluator characteristics on composite score.

(A) Comparison by profession				
Profession	Adjusted Mean (Standard Error)	Mean Difference (95% CI)	*p*-Value	
Doctor	38.83 (0.26)	6.76 (6.26, 7.26)	<0.001	
Nurse	32.06 (0.26)			
(B) Comparison by seniority within each profession				
Profession	Seniority	Adjusted Mean (Standard Error)	Mean Difference (95% CI)	*p*-Value
Doctor	Senior	42.42 (0.37)	7.19 (6.17, 9.21)	<0.001
	Junior	35.23 (0.37)		
Nurse	Senior	32.08 (0.37)	0.05 (−0.98, 1.08)	0.915
	Junior	32.03 (0.37)		

Adjusted means and mean differences were derived from the mixed-effects model, controlling for LLM and use of prompt.

**Table 6:** Qualitative analysis of deficiencies in LLM-generated recommendations with harm assessment and corrective actions.

Scenario	LLM	Identified Deficiency	Potential Harm and Severity	Correct Action Suggested by Expert Panel
9 (Positive AFB smear)	DeepSeek V3	Poor clinical judgment: Recommended empiric TB treatment without considering NTM as a differential diagnosis.	High: Unnecessary exposure of the patient to anti-TB medications and their side effects.	Contact microbiologist or microbiology laboratory to arrange TB-PCR for confirmation.
11 (Screening for *Candida auris*)	DeepSeek V3	Factual inaccuracy: Suggested incorrect screening sites (stool/urine) instead of the standard axilla/groin swabs.	High: A false-negative screening result would lead to the premature discontinuation of contact precautions, creating a significant risk of silent transmission and outbreak of MDRO in the healthcare facility.	Screening using standard axillary and groin swabs.
18 (Router installation)	GPT-4.1	Incompleteness: Lacked a structured risk assessment and failed to ask for critical details (e.g., proximity to immunocompromised patients). Lack of specificity: Did not assign clear responsibilities or define specific environmental controls.	Moderate: Failure to assess risk could lead to construction dust (containing fungal spores like *Aspergillus*) being dispersed in a high-risk area, potentially causing severe invasive infections in immunocompromised patients.	Perform an infection control risk assessment and implement dust control measures appropriate for location and risk level.
21 (*Candida auris* de-escalation)	Gemini 2.5 Pro Exp	Impracticality and lack of conciseness: Overwhelmed the user with dozens of nonessential questions and academic analysis instead of a direct, actionable answer.	Moderate: The confusing and noncommittal response could lead a frontline nurse to make an unsafe decision such as prematurely discontinuing contact precautions based on a single negative test, thereby risking transmission.	Provide a direct ‘yes/no’ answer to the de-escalation question based on hospital policy, followed by a concise rationale.
22 (MDRA isolation from rectal swab)	GPT-4.1	Factual inaccuracy and underestimation of risk: Incorrectly advised that single-room isolation was not required for MDRA, contradicting standard infection control policy.	High: This recommendation directly violates a core principle of MDRO management. Following this advice would create a high probability of nosocomial transmission to other vulnerable patients.	Single room isolation is required for patients colonized with MDRA.
26 (Family member with tuberculosis)	Gemini 2.5 Pro Exp	Lack of conciseness and prioritization: Generated a long, complex document that obscured the key, actionable steps for the healthcare worker, failing to tailor the advice to their immediate needs.	Low: While the information may be technically correct, its poor presentation makes it difficult to use. The user may miss critical advice, leading to anxiety or missed opportunities for personal screening, but the advice itself is not directly harmful.	Prioritize the most critical actions for the healthcare worker and present them in a clear, easily readable list.

Abbreviations: AFB, acid-fast bacilli; LLM, large language model; MDRA, multidrug-resistant *Acinetobacter species*; MDRO, multidrug-resistant organism; PCR, polymerase chain reaction; NTM, nontuberculous mycobacteria; TB, tuberculosis.

## Data Availability

Data are available via a publicly accessible repository at https://doi.org/10.6084/m9.figshare.30149236.v2. The repository contains all anonymized evaluation data and each model’s clarifying questions and recommendations for all case scenarios.

## Supplementary Materials

The following supporting information can be downloaded at: https://www.mdpi.com/article/10.3390/healthcare13202652/s1, Table S1: Thirty clinical infection control scenarios selected for interacting with three LLMs; Table S2: Internal consistency of the evaluation scale; Table S3: Intraclass correlation coefficients (ICCs) for inter-rater reliability across criterion; Table S4: Descriptive statistics of evaluation scale for each large language model; Table S5: Variance components from the linear mixed-effects models for the baseline LLM comparison; Table S6: Pairwise comparisons of estimated marginal mean scores for each criterion; Table S7: Sensitivity analysis using Kruskal–Wallis H test for comparing LLM performance scores; Table S8: Sensitivity analysis of prompting effects, excluding scenarios with generation failures.

## Abbreviations

The following abbreviations are used in this manuscript:

AFB	Acid-fast bacilli
AI	Artificial intelligence
CDC	Centers for Disease Control and Prevention
CI	Confidence interval
CPE	Carbapenemase-producing Enterobacterales
ICO	Infection control officer
ICN(s)	Infection control nurse(s)
ICT	Infection control team
IPC	Infection prevention and control
LLM(s)	Large language model(s)
MDRA	Multidrug-resistant *Acinetobacter* species
MDROs	Multidrug-resistant organisms
MRSA	Methicillin-resistant *Staphylococcus aureus*
NaDCC	Sodium dichloroisocyanurate
NTM	Nontuberculous mycobacteria
SD	Standard deviation
SNO	Senior nursing officer
TB	Tuberculosis
WHO	World Health Organization
