# Supplementary material for: Comparative Evaluation and Performance of Large Language Models in Clinical Infection Control Scenarios: A Benchmark Study
Source: Healthcare (Basel). 2025 Oct 21;13(20):2652. doi: 10.3390/healthcare13202652 (PMC12563182; doi:10.3390/healthcare13202652)
Supplement: Supplementary file 1 [file healthcare-13-02652-s001.zip › healthcare-3894479-supplementary.pdf]

Table S1. Thirty clinical infection control scenarios selected for interacting with three LLMs.

No.	Scenario
1	A person who works as hotel room service staff came to the emergency room to see a doctor after accidentally pricking themselves with a needle while cleaning a room. The doctor asked if they needed a preventive injection. As an infection control nurse, what questions would you like to ask?
2	A patient has developed a rash on their body, and the doctor suspects it might be measles. As an infection control nurse, what questions would you like to ask before giving recommendations?
3	You received a phone call from a nurse who said, "I was assisting a patient with a bronchoscopy when some secretions flew into my eye. What should I do?" As an infection control nurse, what further questions would you like to ask before giving recommendations?
4	You received a phone call from a supporting staff saying, "Today at work, I noticed a lot of condensation in the area where we store the sterile packs. What should I do?" As an infection control nurse, what further questions would you like to ask before giving recommendations?
5	You received a phone call from the hospital: "Our urinal cleaning machine in the ward has been broken for two days. What can I do?" As an infection control nurse, what further questions would you like to ask before giving recommendations?
6	The ward contacted you about a post-natal patient who developed a rash on her body after giving birth. A sample was saved before she was discharged, and the result came back positive for chickenpox. As an infection control nurse, what further questions would you like to ask before giving recommendations?
7	You received a phone call from a nurse in the ward: "There is some yellow liquid dripping from the ceiling in a patient room." As an infection control nurse, what further questions would you like to ask before giving recommendations?
8	You received a call from a ward nurse: "I just admitted a patient who has some bugs crawling on their body." As an infection control nurse, what further questions would you like to ask before giving recommendations?
9	You received a phone call from a ward nurse: "A patient has been here for two weeks, and the AFB smear results from the sputum sample just came back positive." As an infection control nurse, what further questions would you like to ask before giving recommendations?
10	You received a phone call from a ward nurse: "A patient's admission screening results came back positive for vancomycin-resistant enterococci (VRE)." As an

	infection control nurse, what further questions would you like to ask before giving recommendations?
11	You received a phone call with the following question: "The patient was hospitalized in a hospital in mainland China for two weeks a month ago. Should we perform a Candida screening on them? What samples should we collect?" As an infection control nurse, what questions would you like to ask before giving recommendations?
12	You received a call asking: "A patient was admitted with respiratory symptoms, and two days later, we were informed that their brother has chickenpox." As an infection control nurse, what questions would you like to ask before giving recommendations?
13	You received a phone call: "We bought a new healthcare device, and the disinfection method in the manual seems to be different from ours." As an infection control nurse, what questions would you like to ask before giving recommendations?
14	You received a phone call from the ward: "A patient used a bronchoscope that was previously used by another patient and has not been disinfected. What should we do?" As an infection control nurse, what questions would you like to ask before giving recommendations?
15	You received a phone call from the neonatal ward: "We have a comfort glove used on infants, and we're unsure if our disinfection method is appropriate." As an infection control nurse, what questions would you like to ask before giving recommendations?
16	You received a phone call from the occupational therapy department: "We have some feeding tools for children with cleft lip and palate. Is it okay to disinfect them with 140 parts per million of sodium dichloroisocyanurate (NaDCC) and then place them in the steam sterilizer?" As an infection control nurse, what questions would you like to ask before giving recommendations?
17	You received a phone call from the ophthalmology department: "We have some tools used to cover patients' eyes during ophthalmic examinations. How should we disinfect them?" As an infection control nurse, what questions would you like to ask before giving recommendations?
18	You received a phone call from the ward: "We need to install a router for internet connection at the nurse's station. What do we need to prepare?" As an infection control nurse, what questions would you like to ask before giving recommendations?
19	You received a phone call from a nurse at the endoscopy center: "We need to renovate the reprocessing area of the endoscopy center. What should we pay attention to?" As an infection control nurse, what questions would you like to ask before giving recommendations?
20	You received a phone call from the department manager: "The isolation ward with negative pressure rooms requires maintenance, and the ward layout will be reconfigured. How should we plan the new layout?" As an infection control nurse, what questions would you like to ask before giving recommendations?

21	You received a phone call from a nurse in the ward, asking: "If I have a patient labeled as a *Candida auris* contact case, and this patient has one set of screening tests that are negative, may I remove the label?" As an infection control nurse, what questions would you like to ask before giving recommendations?
22	You received a phone call from the ward: "A patient is positive for multidrug-resistant *Acinetobacter* species (MDRA) from their rectal swab. Do they require single room isolation?" As an infection control nurse, what questions would you like to ask before giving recommendations?
23	You received a phone call from the ward: "There is a patient who previously tested positive for *Clostridioides difficile*. When can we discontinue contact precautions?" As an infection control nurse, what questions would you like to ask before giving recommendations?
24	You received a phone call from the ward: "How do we perform decolonization for methicillin-resistant *Staphylococcus aureus* (MRSA)?" As an infection control nurse, what questions would you like to ask before giving recommendations?
25	You received a phone call from the ward: "There is a case of disseminated herpes zoster, but there are no beds available in the airborne infection isolation room. How should we handle this?" As an infection control nurse, what questions would you like to ask before giving recommendations?
26	You received a phone call with the following question: "I am a healthcare worker. My father has been treated for tuberculosis and has been discharged from the hospital. What should we be aware of?" As an infection control nurse, what questions would you like to ask before providing recommendations?
27	You received a phone call from the ward: "We have a patient with influenza A pneumonia. When can we discontinue droplet precautions?" As an infection control nurse, what questions would you like to ask before giving recommendations?
28	You received a phone call from a healthcare worker, asking: "We have a colleague with shingles. Can they still come to work?" As an infection control nurse, what questions would you like to ask before giving recommendations?
29	You received a phone call: "Several colleagues in our department have symptoms of vomiting and diarrhea. Is there anything we should do?" As an infection control nurse, what questions would you like to ask before giving recommendations?
30	You received a phone call from a ward nurse, asking: "A mother has been confirmed with carbapenemase-producing Enterobacterales (CPE) colonization. Can she breastfeed?" As an infection control nurse, what questions would you like to ask before giving recommendations?

Table S2. Internal consistency of the evaluation scale

Criterion	Corrected item-total correlation	Cronbach's alpha if item deleted
Coherence	0.854	0.911
Conciseness	0.775	0.924
Usefulness and relevance	0.875	0.906
Evidence quality	0.742	0.935
Actionability	0.884	0.904

The overall Cronbach's Alpha for the 5-item scale is α = .932 (N = 2,640).

Table S3. Intraclass correlation coefficients (ICC) for inter-rater reliability across criterion

Criterion	ICC (2,16)	95% CI	Interpretation
Coherence	0.411	0.346 - 0.472	Poor
Conciseness	0.815	0.781 - 0.846	Good
Usefulness and relevance	0.478	0.416 - 0.536	Poor
Evidence quality	0.735	0.692 - 0.774	Moderate
Actionability	0.533	0.474 - 0.588	Moderate

Interpretation of ICC: poor (<0.50), moderate (0.50–0.75), good (0.75–0.90), and excellent (>0.90). [1]

Table S4. Descriptive statistics of evaluation scale for each large language model

Criterion	GPT-4.1	DeepSeek V3	Gemini 2.5 Pro Exp
Coherence	7.57 ± 1.56	7.50 ± 1.65	7.23 ± 1.61
Conciseness	7.41 ± 1.53	7.54 ± 1.60	5.91 ± 1.90
Usefulness & relevance	7.31 ± 1.66	7.15 ± 1.81	6.91 ± 1.69
Evidence quality	6.99 ± 1.98	6.65 ± 2.09	6.24 ± 2.05
Actionability	7.49 ± 1.65	7.41 ± 1.75	6.94 ± 1.80
Composite score	36.77 ± 7.53	36.25 ± 8.02	33.22 ± 7.92

All results are expressed in mean ± SD.

Table S5. Variance components from the linear mixed-effects models for the baseline LLM comparison.

Dependent variable	Variance component	Variance estimate	Percent of total variance (%)
Coherence	Evaluator (Between-Group)	1.268	47.8%
	Scenario (Between-Group)	0.040	1.5%
	Residual (Within-Group)	1.343	50.7%
Conciseness	Evaluator (Between-Group)	0.843	29.9%
	Scenario (Between-Group)	0.035	1.2%
	Residual (Within-Group)	1.942	68.9%
Usefulness & relevance	Evaluator (Between-Group)	0.931	30.7%
	Scenario (Between-Group)	0.078	2.6%
	Residual (Within-Group)	2.018	66.7%
Evidence quality	Evaluator (Between-Group)	1.834	43.1%
	Scenario (Between-Group)	0.069	1.6%
	Residual (Within-Group)	2.353	55.3%
Actionability	Evaluator (Between-Group)	1.157	37.8%
	Scenario (Between-Group)	0.057	1.9%
	Residual (Within-Group)	1.851	60.4%
Composite score	Evaluator (Between-Group)	27.145	43.2%
	Scenario (Between-Group)	1.124	1.8%
	Residual (Within-Group)	34.550	55.0%

Table S6. Pairwise comparisons of estimated marginal mean scores for each criterion

**Comparison**	**GPT-4.1 vs. Gemini 2.5 Pro Exp**	**GPT-4.1 vs. DeepSeek V3**	**DeepSeek V3 vs. Gemini 2.5 Pro Exp**
Coherence	0.34 (0.21, 0.48) (p<0.001)	0.07 (-0.06, 0.20) (p=0.523)	0.27 (0.13, 0.41) (p<0.001)
Conciseness	1.50 (1.34, 1.67) (p<0.001)	-0.13 (-0.28, 0.02) (p=0.117)	1.64 (1.47, 1.80) (p<0.001)
Usefulness & relevance	0.40 (0.23, 0.57) (p<0.001)	0.16 (0.00, 0.31) (p=0.048)	0.24 (0.08, 0.41) (p=0.002)
Evidence quality	0.77 (0.59, 0.95) (p<0.001)	0.35 (0.18, 0.51) (p<0.001)	0.43 (0.24, 0.61) (p<0.001)
Actionability	0.56 (0.40, 0.72) (p<0.001)	0.08 (-0.07, 0.23) (p=0.573)	0.48 (0.32, 0.64) (p<0.001)
Composite score	3.58 (2.88, 4.28) (p<0.001)	0.52 (-0.12, 1.17) (p=0.153)	3.06 (2.36, 3.75) (p<0.001)

CI, confidence interval.

P-values are derived from mixed-effects models. P-values have been adjusted for multiple comparisons using the Bonferroni correction.

Table S7. Sensitivity analysis using Kruskal-Wallis H test for comparing LLM performance scores

Criterion	Overall test statistic (H)	Pairwise comparison	Adjusted p-value
Coherence	25.523 (p<0.001)	GPT-4.1 vs. DeepSeek V3	1.000
		GPT-4.1 vs. Gemini 2.5 Pro Exp	<0.001
		DeepSeek V3 vs. Gemini 2.5 Pro Exp	<0.001
Conciseness	364.013 (p<0.001)	GPT-4.1 vs. DeepSeek V3	0.101
		GPT-4.1 vs. Gemini 2.5 Pro Exp	<0.001
		DeepSeek V3 vs. Gemini 2.5 Pro Exp	<0.001
Usefulness & relevance	25.159 (p<0.001)	GPT-4.1 vs. DeepSeek V3	0.488
		GPT-4.1 vs. Gemini 2.5 Pro Exp	<0.001
		DeepSeek V3 vs. Gemini 2.5 Pro Exp	0.001
Evidence quality	60.393 (p<0.001)	GPT-4.1 vs. DeepSeek V3	0.001
		GPT-4.1 vs. Gemini 2.5 Pro Exp	<0.001
		DeepSeek V3 vs. Gemini 2.5 Pro Exp	<0.001
Actionability	49.827 (p<0.001)	GPT-4.1 vs. DeepSeek V3	1.000
		GPT-4.1 vs. Gemini 2.5 Pro Exp	<0.001
		DeepSeek V3 vs. Gemini 2.5 Pro Exp	<0.001

The Kruskal-Wallis H test was used to assess overall differences in scores among the three large language models (LLMs). Where the overall test was significant ($p < 0.05$), post-hoc pairwise comparisons were conducted with a Bonferroni correction for multiple tests.

Table S8. Sensitivity analysis of prompting effects, excluding scenarios with generation failures (N=1440)

			**Large language model**		**p-value**	
		**GPT-4.1**	**DeepSeek V3**	**Gemini 2.5 Pro Exp**	**Effect of prompt**	**Interaction effect**
Mean change in scoring effect of prompt	Coherence	+0.063 (-0.086, 0.212)	-0.013 (-0.162, 0.136)	-0.054 (-0.203, 0.095)	0.987	0.853
	Conciseness	-0.213 (-0.391, -0.035)	-0.096 (-0.274, 0.082)	-0.092 (-0.270, 0.086)	0.136	0.822
	Usefulness & relevance	+0.192 (0.008, 0.376)	-0.004 (-0.188, 0.180)	+0.030 (-0.154, 0.214)	0.418	0.631
	Evidence quality	+1.062 (0.872-1.252)	+0.388 (0.198, 0.578)	+1.108 (0.918, 1.298)	<0.001	0.006
	Actionability	0.075 (-0.100, 0.250)	+0.008 (-0.183, 0.167)	-0.071 (-0.246, 0.104)	0.988	0.807
	Composite score	+1.179 (0.424, 1.934)	+0.267 (-0.488, 1.022)	+0.921 (0.166, 1.676)	0.055	0.647

Values represent the mean change with 95% CI in score when using a structured prompt compared to an open-ended question. P-values show the significance of the main effect of prompting and the interaction effect between prompt type and LLM, as derived from the mixed-effects models.

References

1. Koo, T.K. and M.Y. Li, *A guideline of selecting and reporting intraclass correlation coefficients for reliability research.* Journal of chiropractic medicine, 2016. **15**(2): p. 155–163.
